# Inflammation-Related Markers and Thyroid Function Measures in Pediatric Patients: Is the Grade of Obesity Relevant?

**DOI:** 10.3390/diagnostics11030485

**Published:** 2021-03-09

**Authors:** Ioana Țaranu, Cecilia Lazea, Victoria Creț, Nicoleta Răcătăianu, Mihaela Iancu, Sorana D. Bolboacă

**Affiliations:** 1 Department of Medical Informatics and Biostatistics, Iuliu Hațieganu University of Medicine and Pharmacy, Louis Pasteur Str., No. 6, 400349 Cluj-Napoca, Romania; taranu.ioana@umfcluj.ro (I.Ț.); sbolboaca@umfcluj.ro (S.D.B.); 2 Pediatric Hospital 1, Clinical Emergency Pediatric Hospital, Calea Moților, No. 68, 400370 Cluj-Napoca, Romania; cecilialazea@umfcluj.ro (C.L.); victoria_cret@yahoo.com (V.C.); 3 Department of Pediatrics 1, Iuliu Hațieganu University of Medicine and Pharmacy, Calea Moților, No. 68, 400370 Cluj-Napoca, Romania; 4 Infectious Diseases Clinical Hospital, Integrated Ambulatory-Endocrinology, Moților Str., No.19, 400000 Cluj-Napoca, Romania

**Keywords:** pediatric obesity, differential leukocyte count, inflammation, thyroid function tests

## Abstract

We aimed to investigate the effect of weight status on inflammation-related markers and thyroid function tests in overweight and obese pediatric patients. Children and adolescents diagnosed between January 2017 and January 2019 with overweight or obesity were included in the study. Neutrophil-to-lymphocyte ratio (NLR), platelet-to lymphocyte ratio (PLR) and systemic immune-inflammation index (SII) were calculated for the groups defined according to Body Mass Index (BMI)-for-age *z*-score: overweight (≥1 BMI-for-age *z*-score), obese (≥2 BMI-for-age *z*-score) and severely obese (≥3 BMI-for-age *z*-score). Severely obese patients had significantly higher value of white blood cells (WBC) counts (median = 7.92) compared with overweight patients (7.37, *p* = 0.014). Absolute lymphocyte count was significantly associated with obesity degree in children (Spearman’s Rho coefficient *ρ* = 0.228. *p* = 0.035), whereas absolute polymorphonuclear neutrophils (PMNCs) count was significantly higher in severely obese adolescents than overweight adolescents (overweight: 4.04 vs. severely obese: 5.3 (*p* = 0.029)). In 8.19% of patients an elevated thyroid-stimulating hormone (TSH) level was found, and 3.36% of patients had a low level of free thyroxine with an elevated level of TSH. Total absolute WBC count may be a reliable inflammation-related marker in obese pediatric patients without metabolic syndrome, but needs to be validated in the context of all possible covariates. Subclinical and overt hypothyroidism may develop from an early age in overweight or obese patients.

## 1. Introduction

Obesity is associated with increased mortality in adulthood [1] and the risk of cardiometabolic multimorbidity (defined as the presence of at least two out of type 2 diabetes, coronary heart disease, and stroke) rises from twice in overweight to 15 times in severely obese individuals (WHO class II and III obesity, where WHO = World Health Organization) [2]. Consensus upon definition of cardiometabolic risk (CMR) in children has not been reached [3,4,5], but adiposity, lipid profile, glycaemia, insulin level and blood pressure are common elements of the CMR cluster across studies and higher degrees of obesity are associated to an increase in metabolic risk [6,7]. Longitudinal studies showed that obesity in childhood—defined as BMI-for-age (where BMI = body mass index) and sex above the 95th percentile (International Obesity Task Force (IOTF) references)—increases the CMR in adulthood [8], mainly via inflammation and oxidative stress [9].

In the American pediatric population, based on CDC (Centers for Disease Control and Prevention) thresholds, a new definition for obesity has been recently proposed: class I obesity (≥95th percentile to <120% of the 95th percentile), class II obesity (≥120% to <140% of the 95th percentile or BMI ≥ 35), and class III obesity (≥140% of the 95th percentile or BMI ≥ 40). Following these criteria, the prevalence of severe obesity in American children (≥120% of the 95th percentile) has increased between 1999–2014 from 4% to 6.3% [10].

World Health Organization (WHO) growth charts from 2007 state a threshold of ≥ +1 BMI-for-age *z*-score for overweight, ≥ +2 BMI-for-age *z*-score for obese and ≥ +3 BMI-for-age *z*-score relative to the median for severe obesity [11]. Cole et al. proposed cutoffs for overweight and obesity using country-specific centile curves corresponding to BMI at the age of 18 (IOTF BMI cutoff values) [12].

According to the WHO European Childhood Obesity Surveillance Initiative (COSI), which included data from 21 European countries, 1 in 4 obese children are severely obese based on WHO and IOTF criteria. During the first three COSI rounds of data collection (2007–2013), the prevalence of severe obesity among 4274 school-aged Romanian children was 2.2% [13].

Systemic subclinical inflammation plays a central role in the onset of obesity-driven comorbidities in children and adults [14,15,16]. Novel biomarkers for subclinical inflammation in obesity are tested as cardiovascular risk assessment tools [17,18]. The neutrophil-to-lymphocyte ratio (NLR) and platelet-to lymphocyte ratio (PLR) derived from complete blood cell counts are associated with changes in BMI status in adults [19] and children [20,21]. Neutrophil-to-lymphocyte ratio is emerging as a biomarker for subclinical atherosclerosis in adults and is associated with visceral adiposity excess and pro-inflammatory cytokines release [22]. An increased NLR was found in adults with a high risk of type 2 diabetes (i.e., low insulin sensitivity) [23]. No differences in the NLR were observed in patients with differentiated thyroid cancer and those with benign thyroid nodules [24], but higher values were associated with tumor size, invasion and metastasis [25]. Neutrophil-to-lymphocyte ratio showed elevated values among patients with Hashimoto’s thyroiditis [26,27], but the results regarding the associations with anti-thyroglobulin (TG) antibodies or anti-thyroid peroxidase (TPO) antibodies, thyroid-stimulating hormone (TSH), and free thyroxine (fT4) are still conflicting [26,28,29]. 

The mechanism of inflammation in obesity implies early recruitment of neutrophils followed by infiltration of macrophages [30], this latter step requiring lymphocyte (mainly CD8+ subtype) activation [16]. Accordingly, peripheral neutrophils and lymphocytes counts increase with BMI [31]. Thus, NLR may be a more sensitive indicator of inflammation than differential cell counts, and several studies confirmed that NLR is more increased in obese youth than in normal-weight patients [20,21]. A significant trend of increasing NLR with progressive BMI group in the pediatric population was reported (from a mean value of 1.44 (±1.96 × SD: 1.38–1.49) in normal-weight children to a mean value of 1.74 (±1.96 × SD: 1.64–1.83) in obese children) [32].

Although platelets’ role is primarily associated with hemostasis and thrombosis, these non-nucleated cells have a wide range of functions involving inflammation, immune responses and host defense against pathogens [33,34]. Release of pro-inflammatory chemokines and cytokines from platelet alpha (α) granules and platelet interaction with leukocytes triggers adaptive immune response via dendritic cell maturation and natural killer cells followed by monocyte/macrophage activation, which further affect B and T cell responses [35,36]. Among complex relationships between activated platelets with leukocytes, circulating platelet-neutrophil complexes may be necessary for neutrophil recruitment in inflammatory processes [37]. On the other hand, lymphocytes influence platelet aggregation and secretion [38].

Systemic immune-inflammation index (SII)—defined as a product of peripheral platelet and neutrophil counts divided to lymphocyte counts—and PLR—defined as peripheral platelets and absolute lymphocyte count ratio—were positively associated with BMI in children with morbid obesity (> WHO 99th percentile), and young patients (mean age of 33 ± 7 years) with BMI ≥ 45 kg/m^2^, respectively [39,40].

The above evidence legitimates the use of PLR, NLR and SII as inflammatory markers of subclinical obesity-induced inflammation. Age-specific reference values for these ratios have been proposed [41,42].

Subclinical hypothyroidism (SHT) (or isolated hyper-thyrotropinemia)—defined as a mild elevation of serum TSH concentration and a normal peripheral thyroid hormones concentration—has a prevalence of 1.7% in the pediatric population [43] and is more common in obese pediatric patients than in normal-weight patients (24.3% vs. 12.8%, *p* = 0.002) [44]. Thyroidal and non-thyroidal diseases may induce SHT, among which the most common are Hashimoto’s disease and isolated non-autoimmune hyper-thyrotropinemia, the latter being more prevalent in obese children [45,46]. In adults, NLR is more increased in patients with malignancy than in patients with benign thyroid nodules and is associated with a higher degree of thyroid function loss [47,48].

The objectives of the present study were:

(i) to investigate the effect of weight status defined according to WHO criteria on measures of laboratory tests performed in pediatric patients (as routine evaluation according to the national guideline);
(ii) to evaluate the effect of weight status on inflammation-related markers in pediatric patients; and
(iii) to explore the relationships between the inflammation-related markers and thyroid profile in the overweight or obese pediatric patients.

## 2. Materials and Methods

A cross-sectional study design with retrospective data collection was conducted between January 2017 and January 2019 in two medical services from Cluj-Napoca, namely the Children’s Emergency Hospital and the Ambulatory Unit of Infectious Disease Hospital. Medical records of children aged from 2 to 18 years old diagnosed with overweight or obesity (ICD diagnosis code—E 66.0 or E 66.9) were retrieved for data collection.

We excluded patients with missing data regarding the anthropometric measures, neutrophils or lymphocyte counts. We also excluded patients with syndromic obesity, those who did not meet the WHO criteria for overweight and obesity, data from the second visit of the same patient and patients with acute or chronic known inflammatory diseases (i.e., juvenile arthritis, thyroiditis with hypo- or hyperthyroidism) and infectious disease (acute upper or lower respiratory tract diseases, urinary infections or gastroenteritis, etc.). Patients with leukocytosis, human C-reactive protein serum concentration (CRP) ≥10 mg/dL, positive pharyngeal exudate, positive urine test, or positive stool culture were also excluded.

Height and weight were measured once by stadiometer and mechanical beam scale to ± 0.1 cm and ± 0.1 kg, respectively, after a minimum 8-h fasting period. We collected data for the following characteristics: anthropometric measures (weight and height), inflammation-related markers such as total absolute white-blood cells count (WBC), absolute polymorphonuclear neutrophils count (PMNCs), lymphocytes, absolute peripheral platelets counts, human C-reactive protein (hCRP), liver enzymes: alanine aminotransferase (ALT), aspartate aminotransferase (AST), alkaline phosphatase, gamma-glutamyl transferase (GGT), uric acid, fasting blood glucose and lipid panel (triglycerides, high-density lipoprotein-cholesterol (HDL-c), measures of thyroid function (thyroid-stimulating hormone level (TSH), free thyroxine level (fT4), anti-thyroid peroxidase antibody level (anti-TPO)), fasting blood insulin and morning cortisol levels.

The ancillary laboratory blood tests evaluated in the study are part of the routine evaluation for obesity-related comorbidities in obese children in the Romanian Ministry of Health guideline from 2011 [49].

Blood parameters measurements were performed according to standard guidelines for each technique. Differentiated blood cell count was performed via cytometry, impedance, and colorimetry. Biochemical parameters were measured via spectrophotometry, and hormonal measurements were performed on chemiluminescence. NLR was calculated as a ratio between absolute PMNCs and lymphocyte counts. SII was calculated based on PMNCs, absolute lymphocytes (L) and peripheral platelets (P) counts as PMNCs×L/P. PLR was calculated as the ratio between peripheral platelets (P) and absolute lymphocyte counts.

Blood samples were evaluated in the clinical laboratories of each center. As the normal ranges of blood parameters were slightly different between the two laboratories, each value was interpreted as “normal”, “high”, “low”, “lower border”, or “upper border”. For the “border” categories, we included data with values within ± 0.5 units caliper. The results, expressed as dichotomous variables (i.e., above or under the threshold) were analyzed accordingly, as categorical variables.

WHO guidelines (WHO Child Growth Standards for children aged 0–60 months and the WHO Reference 2007 for the older children and adolescents) were used for defining weight status: overweight, obesity, and severe obesity. AnthroPLus application v1.0.4 was used to compute the *z*-scores for BMI-for-age. We grouped patients as overweight (≥1 BMI-for-age *z*-score), obese (≥2 BMI-for-age—*z*-score), or severely obese (≥3 BMI-for-age *z*-score), respectively. Age was expressed in months both for analysis and BMI-for-age *z*-score computation.

The age classes were defined according to the MESH term in MEDLINE for Pediatric obesity: childhood—between 2 years (24 months) and 11 years and 11 months (143 months)—and adolescence—between 12 years (144 months) and 17 years and 11 months (215 months).

Median values of cell subtypes’ absolute counts were graphically represented according to the following age groups: 2–<6 years, 6–<10 years, 10–<13 years, 13–<15 years, 15–<18 years. These groups follow worldwide hematology reference ranges in pediatrics [50,51] and had been previously reported in studies on Romanian children [52].

### Statistical Analysis

Statistical analysis was performed using Statistica program (Version 13.5, StatSoft, OK, USA). Qualitative nominal variables were reported as absolute and relative frequencies (expressed as %).

Quantitative variables with deviations from Gaussian distribution were presented as median with interquartile interval (IQR: 25–75 percentile) and range values (minimum to maximum values). The arithmetic mean and standard deviation (SD) were used as descriptive measures for quantitative variables that followed Gaussian distribution. The Kolmogorov-Smirnov test was used to investigate if distributions of studied variables followed the Normal Probability Law.

Spearman’s rank correlation coefficient (*ρ*) was used to evaluate the monotonic correlations between quantitative variables while Pearson’s correlation coefficient (*r*) was used to assess linear correlations. Chi-Squared or Fisher’s Exact tests were used to test the bivariate associations between qualitative variables.

For identifying significant differences in distributions of biochemical, hormonal, and inflammation-related characteristics between two independent samples, we used Student *t*-test for independent samples or Mann-Whitney U Test, while for three independent samples, one-way ANOVA or Kruskal-Wallis test were used. In the case of significant differences for multiple groups, we performed the post-hoc analysis with Tukey-HSD test or Dunn’s test.

All statistical tests used in data analysis were two-sided tests, a significant result being achieved if *p*-value < 0.05.

Graphical representations were designed in R statistical software (version 4.03., R Foundation for Statistical Computing, Vienna, Austria).

## 3. Results

### 3.1. Description of the Children and Adolescents Sample

One hundred and seventy-six patients aged from 2 to 18 years were eligible for the study. Thirty-four children were excluded due to missing data regarding neutrophil and lymphocyte counts, a sample size of 142 being included in the analysis. Differences in BMI-for-age *z*-score and laboratory measurements (as routine evaluation in obese children) of all the patients included in the study according to weight status, gender, and age are summarized in Table 1. There were significant differences in the means of BMI *z*-scores between children and adolescents (Student *t*-test, *p* = 0.008), with higher mean values of BMI *z*-scores in children (2.82 ± 1.00 versus 2.33 ± 0.83). The significant differences in BMI *z*-scores were also related to gender (Student *t*-test, *p* = 0.045) with higher mean values in boys (2.79 ± 1.03 versus 2.47 ± 0.80).

Glycemic levels were physiological in all patients (minimum to maximum value: 66 mg/dL–104 mg/dL) and only four patients (3.65%) had both increased ALT and AST levels, with a maximum value of 138 UI/L and 75 UI/L, respectively.

As far as lipid profile is concerned, hypertriglyceridemia was found in 15 (10.56%) patients, among whom 14 had low or low-border HDL-cholesterol levels. A total of 76 patients (53.52%) had low-border levels of HDL-cholesterol (range values: 36–59 mg/dL) and 17 (11.97%) had low HDL-cholesterol levels (below 36 mg/dL). High uric acid levels were found in 17 (26.56%) patients. No cortisol secretion impairment was present in our patients and hyperinsulinemia was found in only seven (4.9%) patients.

Post-hoc analysis showed that HDL-cholesterol mean values were significantly higher in overweight as compared to obese (Tukey’s HSD test, *p* = 0.025) and severely obese (Tukey’s HSD test, *p* = 0.007) children. In addition, there was a negative significant monotonic relationship between HDL-cholesterol and age in the whole sample (Spearman’s Rho coefficient, *ρ* = −0.28, *p* = 0.003).

### 3.2. Distribution of Inflammation-Related Markers Values and Measures of Thyroid Function Amongst Overweight and Obese Children and Adolescents

Differences of inflammation-related marker values (hCRP, total WBC and subtypes (lymphocytes and PMNCs) absolute counts, peripheral platelet count, along with NLR, PLR and SII)) and measures of thyroid function according to weight status, age, and gender groups, are presented in Table 2.

There was a significant difference in distributions of absolute lymphocyte counts and WBC counts between weight groups (Kruskal-Wallis, *p* = 0.040 for lymphocyte and *p* = 0.022 for WBC). Post-hoc analysis showed that severely obese patients had significantly higher value of WBC counts compared with overweight patients (Dunn’s test, adjusted *p* = 0.014). When stratified by age group, a significant relationship between WBC counts and weight status was found in adolescents (Kruskal-Wallis, *p* = 0.027) and children (Kruskal-Wallis, *p* = 0.024).

**Figure 1:**
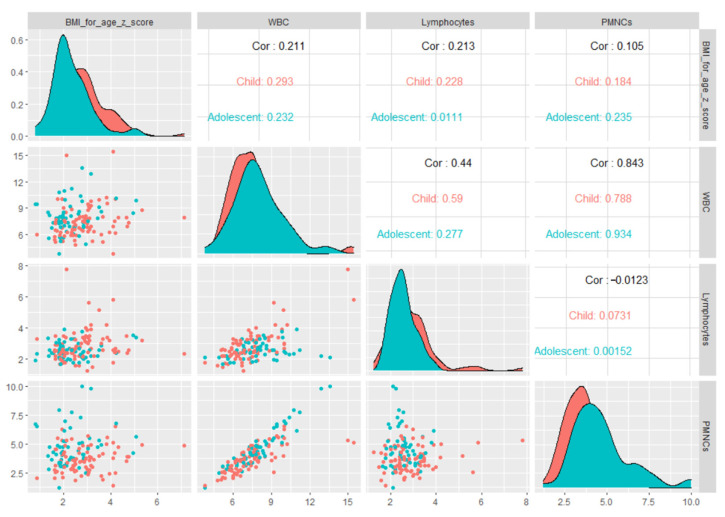
Matrix of scatter plots of correlations between BMI-for-age *z*-scores and inflammation-related markers stratified by age groups.

When we explored the association between WBC and BMI-for-age *z*-scores stratified by age groups (Figure 1), we found a significant positive correlation in children (Spearman’s Rho coefficient *ρ* = 0.293, *p* = 0.006), while for adolescents a positive correlation was found with a tendency toward significance (Spearman’s Rho coefficient *ρ* = 0.23, *p* = 0.081).

The post-test analysis performed to identify differences in WBC subtype counts highlighted that severely obese patients had significantly higher values of absolute lymphocyte count than overweight patients (severely: 2.70 (IQR: 2.23–2.89) vs. overweight: 2.47 (IQR: 2.16–2.81), Dunn’s test, adjusted *p* = 0.022). When stratified by age group, the relationship between peripheral white cell subtypes counts and weight status had a tendency toward statistical signification for children (Kruskal-Wallis, *p* = 0.056) and it was not significant for adolescents (Kruskal-Wallis, *p* = 0.849). When stratified by age groups, it was only in children that absolute lymphocyte count was significantly correlated to BMI-for-age *z*-score (Spearman’s Rho coefficient *ρ* = 0.228, *p* = 0.035). We found no significant difference in distributions of PMNC values and peripheral platelet count between the three weight groups (*p* > 0.05; see Table 2). In adolescents, absolute PMNC count distributions were significantly different between overweight and severely obese patients (overweight: 4.04 (IQR: 3.33–4.71) vs. severely: 5.3 (IQR: 4.68–5.6), Dunn’s test, adjusted, *p* = 0.029) while for the children subgroup we did not find a significant difference in absolute PMNC count distribution by weight status (Kruskal-Wallis, *p* = 0.258).

**Figure 2:**
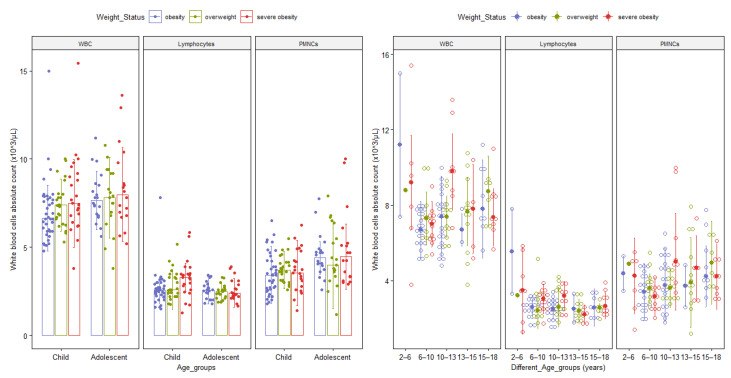
Bar and Dot plots with median and interquartile range of differential white cell subgroups by age group and obesity class. The upper bound of each age group is not included in the range. Abbreviations: WBC = total absolute white blood cells count; PMNCs = absolute polymorphonuclear neutrophils absolute counts.

The white blood cell (WBC, lymphocytes, PMNCS) values stratified by different age groups are also described in Figure 2.

We found no significant differences in mean values of NLR, SII and PLR between the three weight groups (one-way ANOVA, *p* > 0.05; Table 2). When we performed the stratified analysis by age groups, NLR, SII and PLR mean values were not significantly different between the three weight subgroups, neither for children, nor for adolescents (Kruskal-Wallis test, *p* > 0.05). In addition, when we explored the association between these biomarkers and BMI-for-age *z*-scores, we found no significant correlation between NLR and SII with BMI-for-age *z*-score (Spearman’s Rho coefficient, *ρ* = −0.06, *p* = 0.45), whereas PLR was significantly correlated with BMI-for-age *z*-score (Spearman’s Rho coefficient, *ρ* = −0.17, *p* = 0.03). In the whole sample, the NLR and SII values increased with age (Spearman’s Rho coefficient, *ρ* = 0.34, *p* < 0.0001, and *ρ* = 0.24, *p* = 0.003, respectively) with significantly different NLR mean values among adolescents as compared to children (Student-t test, *p* < 0.001).

No significant differences in mean values of NLR, PLR and SII were observed between boys and girls (Student-t test, *p* > 0.05; Table 2).

We found no significant differences in mean values of fT4 by weight status neither in children (one-way ANOVA, *p* = 0.452, mean ± SD for overweight = 0.99 ± 0.16, mean ± SD for obese = 0.96 ± 0.18, mean ±SD for severe obese = 1.02 ± 0.21), nor in adolescents (one-way ANOVA, *p* = 0.221, mean ± SD for overweight = 0.96 ± 0.19, mean ± SD for obese = 0.90 ± 0.12, mean ± SD for severe obese = 0.99 ± 0.09). We found a low positive correlation between fT4 level and BMI-for-age *z*-score (Pearson’s correlation coefficient, *r* = 0.18, *p* = 0.046). No significant correlation with age was found (Spearman’s Rho coefficient *ρ* = −0.12, *p* = 0.192). A tendency toward statistical significance was noticed for difference in TSH mean values by weight status in children (one-way ANOVA, *p* = 0.074, mean ± SD for overweight = 2.58 ± 1.27, mean ± SD for obese = 3.80 ± 2.17, mean ± SD for severe obese = 3.09 ± 1.39), but not for adolescents (one-way ANOVA, *p* = 0.774, mean ± SD for overweight = 2.87 ± 1.38, mean ± SD for obese = 2.673 ± 1.40, mean ± SD for severe obese = 2.50 ± 1.51).

When we interpreted anti-TPO, TSH and fT4 blood levels according to laboratory normal reference limits (as “normal”, “low”, “high” and “upper limit”), we found no significant association with weight status (Fisher’s-Exact test, *p* > 0.05). In 10 (8.19%) patients an elevated TSH as a unique change was found and only four (3.36%) patients had a low level of fT4 and an elevated level of TSH. Positive thyroid antibodies were found in three patients.

### 3.3. Correlations between Inflammation-Related Markers and Thyroid Function Measures

We found a significant positive correlation between absolute peripheral platelets count and fT4 value when the whole sample was evaluated (Spearman’s Rho coefficient, *ρ* = 0.29, *p* = 0.001; Table 3). A positive correlation between absolute WBC count and fT4 level with a tendency to statistical significance was also found, whereas when the correlation between WBC subtypes and fT4 was considered, we found a significant positive correlation with absolute lymphocyte count with a tendency to significance (Table 3).

## 4. Discussion

No considerable differences in NLR, SII and PLR with different weight status were found in our study. The main cell subtype counts that changed were lymphocyte and polymorphonuclear neutrophils. The absolute white blood cells count varied in close relation to different age groups according to weight status. In addition, absolute WBC and PMNC counts showed higher values with advancing age group in overweight pediatric patients (see Figure 1). Considering that the severely obese subgroup consisted mainly of children and that peripheral lymphocyte count—significantly higher than in the overweight subgroup (as shown in Table 2)—increased in an age-independent manner, we might assume that their increase occurs earlier in severe obesity than in obesity and overweight subgroup. Our results are partially consistent with those previously reported on an American pediatric population [53].

Contrarily, the polymorphonuclear neutrophils count changes later in life in both severely obese pediatric patients and patients with a lower degree of obesity (see Table 2). Similar variations of differential white blood cell count in normal-weight healthy pediatric patients have been acknowledged in other studies and reference values have already been proposed (i.e., in early childhood polymorphonuclear neutrophils count is the lowest and lymphocyte count is the highest and they change in an opposite direction with time) [54,55,56,57].

Macrophages M2 are the most abundant innate immune cells activated in obesity [58], but recent studies showed that neutrophil activation also mediates subclinical inflammation in obesity mainly via elastase and myeloperoxidase secretion [59,60]. In both children and adults, neutrophil count was associated with obesity degree, even in the absence of metabolic syndrome [61,62].

We found a significant positive monotonic relation of WBC counts with BMI-for-age *z*-score in children and a positive correlation with a tendency toward significance in adolescents. Similar trends in WBC count elevation with age have been reported in healthy European adolescents with a higher increase rate in overweight adolescents [54]. Our findings on differences in WBC count between weight groups agree with reported results of higher WBC count levels in obese than overweight or overweight than normal weight pediatric patients [21,63]. WBC count in obese adults and adolescents was shown to be closely related to leptin concentration, a hormone secreted by adipose cells: leptin serum concentration is associated to WBC counts [64,65,66]. In vivo experiments on murine models revealed that myeloid proliferation is enhanced by high leptin concentration in obesity [67,68]. 

Our results regarding differential peripheral white blood cell counts indicate an age-independent degree of dynamic relation with obesity that may be further investigated in prospective studies including pediatric patients. The accessibility of complete blood count in pediatrics and the need for markers for evaluating the risk of obesity-driven complications is an incentive to search for new methods which may serve this scope. White blood cell count may provide support for the clinical use of markers in childhood obesity.

Early age onset of overweight is associated with a higher risk of morbidity of coronary heart disease than late-onset overweight [69] and childhood obesity is associated with a higher prevalence of cardiovascular risk factors during adulthood [70]. More than half of our patients had low-border level or low HDL-cholesterol level—a known cardiovascular risk factor in adults and a component of metabolic syndrome in children [71] —leading to a significant difference between weight groups (Table 1). Subclinical inflammation is the linking mechanism of obesity with metabolic syndrome and elevated WBC count is a biomarker of inflammation in obese adults [72,73] and pediatric patients [74,75] with metabolic syndrome. However, inconsistent results were reported regarding association of NLR with the severity of metabolic syndrome and risk of type 2 diabetes mellitus in adults [73,76,77]. Fasting glycaemia and fasting insulinemia in obese children were not significantly associated with the degree of obesity or the risk of progression to type 2 diabetes [78,79,80]. In this sense, performing OGTT (oral glucose tolerance test) or proinsulin levels and proinsulin/insulin ratio were more significant for the assessment of metabolic impairment [78,79,80].

One possible cause for children with severe obesity included in our study having slightly lower values of fasting glucose compared to those with overweight/moderate obesity (Table 1) could be an imbalance between insulin resistance and insulin sensitivity, with fasting hyperinsulinemia and episodes of reactive, functional hypoglycemia. In addition, evidence has also shown that some obese children have episodes of nocturnal hypoglycemia, while others have episodes of hyperglycemia without any obvious cause, more likely to be associated with altered secretion of other hormones/polypeptides involved in glycemic control [81,82].

Regarding the measures of thyroid function, we found no significant relation with inflammation-related markers (see Table 3). However, we observed significant correlations between fT4 level and absolute WBC and peripheral platelet counts (Table 3). Interpretation of changes in fT4 level should carefully consider the possibility of inter-assay interferences known to affect TSH, fT4 and fT3 levels when measured on immunoassay platforms [83]. To avoid misinterpretation, only concordant changes were taken into consideration (i.e., low/lower normal limit TSH level with normal/high fT4 level—overt or subclinical hypothyroidism) in our analysis. Another common pitfall is the overlooking the adaptive changes during inflammatory moderate to severe acute or chronic illness—the “non-thyroidal illness” [84]. In 8.19% of patients, we found changes suggestive of subclinical hypothyroidism and 3.36% patients had changes of overt hypothyroidism. Similarly, a higher prevalence of elevated TSH level than positive antibody dysfunction was reported in the literature [46]. A possible explanation may be the obesity related TSH elevation in the former and primary thyroid disease in the latter [46,85]. The TSH serum levels in overweight and obese children and adolescents showed a significant reduction with the decrease of BMI (from 5.4 ± 1.4 to 4.9 ± 1.5 mU/L, *p* < 0.0001) without significant changes in serum level of fT4 (*p* = 0.210) and a tendency to a significant increase in NLR (*p* = 0.08) [86]. In obesity, subclinical inflammation leads to tissue resistance to TSH followed by a high blood level of this hormone. This mechanism can be explained by the role of inflammatory cytokines upon the iodide uptake activity of thyroid cells [87,88], leptin inhibition of TSH-induced function [89] or induced thyroid autoimmunity via T-regulatory cell function [90,91].

The present results with respect to thyroid dysfunction in relation to the subclinical inflammation in childhood obesity provide support for prospective studies that may elucidate whether it plays a causative or consequential role.

### Study Limitations and Further Studies

As our subgroups were not comparable for age, we adjusted the analysis by performing stratified analysis. However, some limitations of our study should be acknowledged. The main limitation is related to the design of the experiment. The retrospective collection of data does not assure that all eligible patients are evaluated since missing data in medical records cannot be prevented. The absence of normal controls is another drawback of our study that emerged from the retrospective collection of data. Inclusion of children in medical studies is still challenging and, in the absence of electronic health records to capture medical visits for routine check-ups and access to healthy controls, their inclusion in our study was not possible. In the prospective study currently conducted by our team with a focus on new inflammatory markers in childhood obesity we overcome this problem by including a control group in the analysis. Furthermore, a retrospective collection of data does not allow to control for covariates that could be related to observed changes in the reported blood measurements. Cigarette smoking status (passive or active), ethnicity, low-birth weight, medication use within 24 h, and organic pollutants may considerably influence the results of this analysis [92,93,94]. The control of covariates could be achieved by epidemiological longitudinal populational studies. Puberty status also may be important as it is known that normal ranges of WBC increase until puberty and decrease in post-pubertal patients [54]. All the above-mentioned limitations could be eliminated in a prospective longitudinal study. The inclusion of eligible participants from two different healthcare units is also a limitation of our study. To withdraw this limitation, we reported some quantitative measurements as qualitative data according to each laboratory reference for -age and -gender. The limited number of investigated patients along with the absence of a control group limit the generalizability of the reported results. 

The WBC, as a routine laboratory measurement, could be used as an inflammation related marker in paediatric obesity that could be useful in identification of changes prior to the onset of metabolic syndrome. Identification of the cut-off point will bring the evidence into clinical daily practice but the determination of the cut-off values must be done in populational studies with an appropriate inclusion of participants in terms of stages of obesity, stratified by age (children vs. adolescents) and gender, in the presence of healthy controls.

Nonetheless, a real advantage of studying blood cell parameters in children is that potential comorbidities that may influence them may be more easily ruled out than in adults (i.e., neoplasms, diabetes, and cardiovascular disease). Therefore, the identification of a marker-based on routine blood cell parameters able to characterize the obesity status in children and adolescents is of interest.

## 5. Conclusions

Total absolute white-blood cells count (WBC) proved to be a reliable inflammation-related marker in pediatric obesity prior to the onset of metabolic syndrome, but this result needs to be validated in the context of all possible covariates. The changes in absolute lymphocyte and polymorphonuclear neutrophils counts with age may influence absolute WBC count’s role in reflecting subclinical inflammation.

Laboratory changes suggestive of subclinical and overt hypothyroidism indicate an obesity-driven thyroid dysfunction, possibly inflammation-mediated.

## Figures and Tables

**Table 1:** BMI-for-age *z*-score, biochemical and hormonal measurements by weight status, gender and age.

Variables	Age Groups			Gender Groups			WHO Weight Status			
	Children (*n*_1_ = 85)	Adolescents (*n*_2_ = 57)	Stat. (*p*)	Boys (*n*_1_ = 69)	Girls (*n*_2_ = 73)	Stat. (*p*)	Overweight (*n*_1_ = 44)	Obesity (*n*_2_ = 57)	Severe Obesity (*n*_3_ = 41)	Stat. (*p*)
AST (U/L) ^a^ Median (Q1-Q3) Min–Max	29 (25–33) 17–75	23 (18–26) 14–56	4.88 (<0.001)	29.5 (23–31) 14–75	26 (22–31) 14–50	0.78 (0.434)	25 (22–29) 14–41	27 (22–31) 14–75	28 (23.5–35) 14–71	4.06 (0.132)
ALT (U/L) ^a^ Median (Q1-Q3) Min–Max	21 (17–27) 8–139	20 (15–28) 8–91	1.00 (0.315)	21 (17–28.5) 8–139	20 (16–26) 8–75	0.98 (0.323)	18.5 (14–24) 9–75	20.0 (17–31) 8–139	22 (17–27) 8–109	4.23 (0.120)
Alkaline phosphatase (U/L) ^b^ Mean ± SD	263.4 ± 72.9	171.2 ± 97.8	−4.29 (<0.001)	256 ± 59.7	199.9 ± 114.1	2.48 (0.014)	231 ± 91.5	229 ± 111.7	224.9 ± 76.3	0.03 (0.973)
GGT (IU/L) ^c^ Median (Q1-Q3) Min–Max	14 (10.5–18.5) 2–72	14 (11–20) 0–152	−0.09 (0.93)	16 (11–23) 0–59	12 (10–17) 2–152	2.35 (0.018)	13 (0–152) 9–17	14.5 (11–19) 2–59	14 (11–21) 5–72	1.39 (0.499)
Glycemia (mg/dL) ^d^ Mean ± SD	81.5 ± 7.2	84.8 ± 9.3	2.33 (0.021)	84.6 ± 8.0	81.1 ± 8.2	2.44 (0.015)	86.5 ±8.2	82.6 ± 8.0	79.3 ± 7.3	8.44 (<0.001)
Uric acid ^b^ Mean ± SD	5.1 ± 0.9	5.5 ± 1.2	1.58 (0.118)	5.4 ± 1.3	5.1 ± 0.9	1.29 (0.201)	4.9 ± 0.7	5.4 ± 1.1	5.3 ± 1.3	0.92 (0.404)
Triglycerides (mg/dL) ^e^ Median (Q1-Q3) Min–Max	79 (59–122) 27–248	92.5 (72–124) 39–223	−1.45 (0.155)	85 (59–142) 27–248	83 (68–108) 42–241	0.20 (0.839)	81 (60–124) 29–241	85 (70–118) 27–236	88 (60.5–123) 40–248	0.59 (0.744)
HDL-c (mg/dL) ^f^ Mean ± SD	49.3 ± 11.4	44.7 ± 11.5	−2.16 (0.033)	46.9 ± 11.3	7.9 ± 12.0	−0.41 (0.681)	52.7 ± 12.5	45.9 ± 10.4	4.6 ± 10.8	5.41 (0.006)
Insulinemia (µIU/mL) ^g^ Median (Q1-Q3) Min–Max	11.1 (6–19) 3.3–61	14.6 (11–22) 0.0–5.9	−2.51 (0.012)	13.9 (9–19) 3.3–67.9	11.5 (9–10) 3.3–61	0.40 (0.685)	9.1 (7–13) 3.3–61	18.2 (13–24) 4.3–67.9	12.1 (9–19) 4.5–46.5	14.54 (0.000)
Cortisolemia (µg/dL) ^h^ Median (Q1-Q3) Min-Max	9.2 (6–13) 2.6–24.7	9.4 (8–12) 3.8–24	−0.74 (0.454)	9.4 (6–12) 2.6–24.7	9.1 (7–15) 3.8–24	−0.51 (0.609)	9.3 (7–16) 4.2–24.7	9.4 (7–12) 3.8–16.3	9.2 (7–13) 2.6–19.2	0.28 (0.869)

Stat. = Test statistics; ^a^ complete case data *n* = 137; ^b^ complete case data *n* = 64; ^c^ complete case data *n* = 98; ^d^ complete case data *n* = 135; ^e^ complete case data *n* = 133; ^f^ complete case data *n* = 116; ^g^ complete case data *n* = 97; ^h^ complete case data *n* = 80; *p*-values obtained from Student-*t* test or Mann Whitney test, one-way ANOVA or Kruskal-Wallis tests; level of significance was set at 0.05; bold values denoted significant test results; Abbreviations: SD: standard deviation, Q1: first quartile (25th percentile); Q3: third quartile (75th percentile); Min: minimum value; Max: maximum value; ALT = alanine aminotransferase; AST = aspartate aminotransferase; GGT = gamma-glutamyl transferase; HDL-c = high-density lipoprotein-cholesterol.

**Table 2:** Inflammation-related markers and thyroid function measures by weight status, age and gender.

Variables	WHO Weight Category				Age Groups			Gender Groups		
	Overweight (*n*_1_ = 44)	Obesity (*n*_2_ = 57)	Severe Obesity (*n*_3_ = 41)	Stat. (*p*)	Children (*n*_1_ = 85)	Adolescents (*n*_2_ = 57)	Stat. (*p*)	Boys (*n*_1_ = 69)	Girls (*n*_2_ = 73)	Stat. (*p*)
WBC (×10^3^/µL) Median (Q1–Q3) Min–Max	7.2 (5.8–7.9) 3.8–10.8	7.4 (6.6–8) 4.8–15	7.9 (6.9–9.3) 3.8–15.4	7.59 (0.022)	7.2 (6.2–8) 3.8–15.4	7.8 (6.8–9.1) 3.8–13.6	−2.25 (0.024)	7.4 (6.5–8.2) 4.8–15.4	7.4 (6.4–8.5) 3.8–15	−0.24 (0.812)
Lymphocytes (×10^3^/µL) Median (Q1–Q3) Min–Max	2.5 (2.1–2.9) 1.7–3.5	2.6 (2.2–3.2) 1.6–7.8	2.7 (2.4–3.4) 1.3–5.9	6.4 (0.040)	2.7 (2.4–3.3) 1.3–7.8	2.5 (2.1–2.8) 1.7–3.9	2.14 (0.032)	2.6 (2.2–3.2) 1.5–5.9	2.6 (2.2–3.1) 1.3–7.8	0.66 (0.509)
PMNCs (×10^3^/µL) Mean ± SD	3.9 ± 1.4	4.0 ± 1.5	4.2 ± 1.5	0.69 (0.500)	3.6 ± 1.1	4.7 ± 1.7	4.37 (<0.001)	4.0 ± 1.6	4.0 ± 1.3	−0.01 (0.991)
Peripheral platelet (×10^3^/µL), Mean ± SD	307.3 ± 65.7	309.2 ± 53.3	302.7 ± 80.1	0.12 (0.887)	314.6 ± 0.7	294.9 ± 54.9	−1.77 (0.078)	312.2 ± 69.9	301.6 ± 60.8	0.97 (0.335)
hCRP (mg/dL) ^a^ Median (Q1–Q3) Min–Max	0.2 (0.1–0.3) 0.0–1.6	0.4 (0.2–0.4) 0.0–0.8	0.31 (0.1–0.4) 0.1–5.9	1.92 (0.382)	0.3 (0.1–0.4) 0.0–1.9	0.3 (0.2–0.4) 0.0–5.9	−0.67 (0.501)	0.3 (0.13–0.4) 0.0–5.9	0.3 (0.1–0.4) 0.0–2.0	−0.19 (0.843)
NLR Mean ± SD	1.6 ± 0.7	1.6 ± 0.8	1.6 ± 0.8	0.04 (0.962)	1.4 ± 0.6	1.9 ± 0.8	4.49 (<0.001)	1.6 ± 0.8	1.6 ± 0.7	−0.16 (0.874)
PLR Mean ± SD	127.7 ± 40.7	119.8 ± 31.1	111.6 ± 50.0	1.69 (0.187)	120.0 ± 46.3	119.8 ± 30.1	−0.03 (0.978)	121.0 ± 40.6	118.8 ± 40.6	0.31 (0.753)
SII Mean ± SD	6876±2749	6399 ± 2389	5943 ± 3470	1.14 (0.322)	6083 ± 3138	6911 ± 292	1.709 (0.089)	6443 ± 2918	6389 ± 2803	0.11 (0.909)
fT4 (ng/dL) ^b^ Mean ± SD	1.0 ± 0.2	0.9 ± 0.2	1.0 ± 0.2	2.6 (0.075)	1.0 ± 0.2	0.9 ± 0.2	−1.65 (0.101)	1.0 ± 0.2	1.0 ± 0.2	−0.50 (0.613)
TSH (µIU/mL) ^c^ Mean ± SD	2.8 ± 1.3	3.3 ± 2.0	3.0 ± 1.4	1.06 (0.349)	3.2 ± 1.7	2.7 ± 1.4	−1.79 (0.076)	2.7 ± 1.5	3.3 ± 1.7	−2.05 (0.040)
anti-TPO (IU/mL) ^d^ Median (Q1–Q3) Min–Max	1.8 (0.8–22.6) 0.1–810	0.8 (0.5–13.4) 0.0–438.4	1.5 (0.6–14.8) 0.0–96.8	2.57 (0.277)	1.0 (0.5–12.5) 0.01–47	2.55 (0.8–19.7) 0.2–810	2.10 (0.035)	1.3 (0.6–12.5) 0.0–46	1.25 (0.7–22.8) 0.01–810	−1.36 (0.173)

Stat. = Test statistics; ^a^ complete case data *n* = 53; ^b^ complete case data *n* = 119; ^c^ complete case data *n* = 122; ^d^ complete case data *n* = 91, we excluded 16.6% values that were reported as qualitative results by the laboratory (under 10 IU/mL); *p*-values obtained from Student-*t* test or Mann Whitney test, one-way ANOVA or Kruskal-Wallis tests; level of significance was set at 0.05;bold values denoted significant test results; Abbreviations: SD: standard deviation, Q1: first quartile (25th percentile); Q3: third quartile (75th percentile); Min: minimum value; Max: maximum value; WBC = total absolute white-blood cells count; PMNCs = absolute polymorphonuclear neutrophils count; hCRP = human C-reactive protein; NLR = neutrophil-to-lymphocyte ratio; PLR = platelet-to lymphocyte ratio; SII = systemic immune-inflammation index; fT4 = free thyroxine level; TSH = thyroid-stimulating hormone level; anti-TPO = anti-thyroid peroxidase antibody level.

**Table 3:** Spearman correlation matrix between inflammation-related markers and thyroid function measures.

Variables	Char.	fT4 (ng/dL)	TSH (µIU/mL)	Anti-TPO (IU/mL)	WBC (×10^3^/µL)	Lymphocytes (×10^3^/µL)	PMNCs (×10^3^/µL)	Peripheral Platelet (×10^3^/µL)	hCRP (mg/dL)	NLR	PLR
WBC (×10^3^/µL)	*ρ*	0.18	−0.05	0.14							
	*n*	119	122	91							
	p*	0.051	0.559	0.177							
Lymphocytes (×10^3^/µL)	*ρ*	0.16	0.05	−0.02	0.44						
	*n*	119	122	91	142						
	p*	0.090	0.571	0.867	<0.001						
PMNCs (×10^3^/µL)	*ρ*	0.11	-0.11	0.13	0.84	-0.01					
	*n*	119	122	91	142	142					
	p*	0.255	0.247	0.225	<0.001	0.885					
Peripheral platelet (×10^3^/µL)	*ρ*	0.29	−0.14	−0.05	0.03	0.04	0.03				
	*n*	119	122	91	142	142	142				
	p*	0.001	0.113	0.669	0.694	0.652	0.721				
hCRP (mg/dL)	*ρ*	−0.34	0.11	−0.28	−0.06	0.09	−0.11	0.06			
	*n*	38	39	22	53	53	53	53			
	p*	0.038	0.502	0.204	0.686	0.506	0.416	0.683			
NLR	*ρ*	−0.05	−0.11	0.10	0.35	−0.62	0.75	−0.01	−0.08		
	*n*	119	122	91	142	142	142	142	53		
	p*	0.562	0.211	0.371	<0.001	<0.001	<0.001	0.934	0.553		
PLR	*ρ*	0.04	−0.14	−0.04	−0.35	−0.75	0.01	0.58	−0.01	0.47	
	*n*	119	122	91	142	142	142	142	53	142	
	p*	0.674	0.135	0.711	<0.001	<0.001	0.937	<0.001	0.926	<0.001	
SII	*ρ*	0.01	−0.15	0.03	−0.09	−0.77	0.33	0.44	−0.05	0.76	0.92
	*n*	119	122	91	142	142	142	142	53	142	142
	p*	0.947	0.102	0.800	0.271	<0.001	<0.001	<0.001	0.719	<0.001	<0.001

Char. = characteristics; p* = p-value; *n* = complete case data; level of significance was set at 0.05; bold values denoted significant test results; WBC = total absolute white-blood cells count; PMNCs = absolute polymorphonuclear neutrophils count; hCRP = human C-reactive protein; NLR = neutrophil-to-lymphocyte ratio; PLR = platelet-to lymphocyte ratio; SII = systemic immune-inflammation index; fT4 = free thyroxine level; TSH = thyroid-stimulating hormone level; anti-TPO = anti-thyroid peroxidase antibody level.

## Data Availability

The raw data presented in this study are part of a PhD study can be obtained upon reasonable request addressed to Ioana Țaranu (taranu.ioana@umfcluj.ro).
